# Circannual Rhythms Affect the Bioavailability of Phenolic Compounds from Grape Seed Proanthocyanidins Extract Differently in Healthy and Obese Fischer 344 Rats

**DOI:** 10.1021/acs.jafc.5c03952

**Published:** 2025-09-26

**Authors:** Iván Escobar-Martínez, Verónica Arreaza-Gil, Anna Arola-Arnal, Begoña Muguerza, Miquel Mulero, Manuel Suárez, Cristina Torres-Fuentes

**Affiliations:** † 16777 Universitat Rovira i Virgili, Departament de Bioquímica i Biotecnologia, Nutrigenomics Research Group, 43007 Tarragona, Spain; ‡ Nutrigenomics Research Group, Institut d’Investigació Sanitària Pere Virgili (IISPV), C/Marcel·lí Domingo 1, 43007 Tarragona, Spain; § Center of Environmental Food and Toxicological Technology (TecnATox), C/Marcel·lí Domingo 1, 43007 Tarragona, Spain

**Keywords:** bioavailability, phenolic compounds, circannual rhythms, GSPE, Fischer 344 rats

## Abstract

(Poly)­phenols are plant-derived bioactive molecules that are associated with several health benefits. Therefore, it is essential to study the factors that affect the bioavailability of these phenolic compounds. Recently, circannual rhythms have been identified as one of the factors that may affect the bioavailability of these compounds. Hence, this study evaluates the impact of circannual rhythms on grape seed proanthocyanidins extract (GSPE) bioavailability in healthy and obesogenic contexts. Male Fischer 344 rats, fed standard (ST) or cafeteria (CAF) diets, were housed under different photoperiod conditions (6, 12, or 18 h of light per day) during 9 weeks and an oral dose of GSPE (25 mg/kg) was daily administered for the last 4 weeks. Serum GSPE-derived metabolites were then quantified by HPLC-ESI-MS/MS. A higher bioavailability was observed in rats exposed to a 12 h photoperiod and fed ST diet. However, this pattern was altered in CAF-fed rats, suggesting an attenuated influence of photoperiod under obesogenic conditions. These findings contribute to a better understanding of the complex relationships between diet, photoperiod, and serum metabolites.

## Graphical abstract

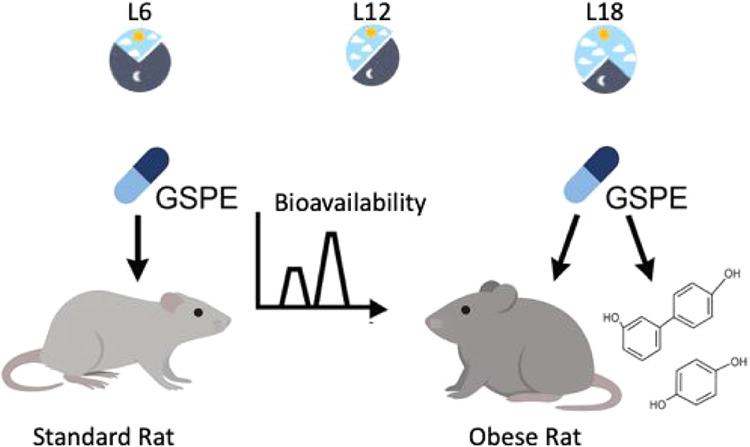

## 1 Introduction

Phenolic compounds, widely known as (poly)­phenols, constitute a group of natural chemical compounds found in vegetables, fruits, grains, and their derived beverages.^1,2^ These compounds are synthesized by most plants in response to stress.^2^ (Poly)­phenols can range from simple molecules to high molecular weight polymers and can be classified into two main groups: flavonoids and nonflavonoids.^3^ In turn, flavonoids can be classified into different subclasses based on the degree of oxidation in the heterocyclic ring, including flavan-3-ols, flavonols, flavones, isoflavones, flavanones and anthocyanidins.^4^ Nonflavonoids include phenolic acids, lignans, stilbenes, tannins, and xanthones.^1^ Consumption of these compounds has been associated with several beneficial effect in cardiovascular, metabolic disorders and certain cancers for example, colorectal, via modulation of inflammation, epigenetics and gut microbiota.^1,5–8^

In order to understand the full spectrum of benefits offered by (poly)­phenols, it is necessary to explore the concept of bioavailability. This term expresses the fraction of ingested nutrient or bioactive compound that reaches the systemic circulation and is ultimately utilized.^9,10^ This intricate process of absorption, distribution, metabolization and excretion of (poly)­phenolic compounds takes place through a complex net of biological interactions that influence the degree to which these compounds can exert their beneficial effects.^9,10^

(Poly)­phenols are commonly found in dietary sources as esters, glycosides or polymeric forms that are not directly absorbed.^11^ In this regard, it has been estimated that only a small fraction of the consumed (poly)­phenols is absorbed within the small intestine, constituting about 5–10%. The others, reach the colon, where undergo extensive microbial transformations.^12^ After absorption, phenolic compounds and their resulting metabolites are transported through the systemic circulation to the different tissues and organs. Within these tissues, they are identified as foreign substances, or xenobiotics, and subsequently undergo a series of comprehensive phase II reactions. These reactions, involving glucuronidation, sulfation, and methylation, are catalyzed by specific enzymes, including uridine 5′-diphospho-glucuronyltransferase (UGTs), sulphotransferases (SULTs), and catechol-*O*-methyltransferase (COMT). Furthermore, these metabolites may undergo recirculation into the intestine through the enterohepatic cycle. Ultimately, the journey of these metabolites, which are transported via the systemic circulation, are excreted through the kidneys via urine. For (poly)­phenols that remain unabsorbed, they are excreted through faeces.^12,13^

The bioavailability and metabolism of these bioactive substances are subject to a multitude of factors. In fact, it has been shown that both external and internal elements, such as (poly)­phenol structure,^14^ environmental conditions,^14^ sex,^15^ age,^16^ gut microbiota-composition,^12,17^ and the specific animal strain,^18^ affect the bioavailability of phenolic compounds. In addition to these factors, circadian and seasonal rhythms have emerged as a key potential modulators of (poly)­phenols bioactivity.^19^ This effect of biological rhythms in (poly)­phenols bioactivity may be due to alterations in their bioavailability. Indeed, we have recently demonstrated how the time of administration can influence the bioavailability of phenolic compounds in both healthy and obese rats.^20^ Moreover, we have also recently shown that exposure to different photoperiods, which mimics different seasons, leads to changes in the bioavailability of these compounds in obese rats and that gut microbiota may be playing an important role.^17^ However, the impact of biological rhythms on (poly)­phenols bioavailability, especially in the case of circannual rhythms, has not been sufficiently investigated yet and more studies are needed. Thus, the aim of this study was to evaluate whether circannual rhythms affect serum bioavailability of phenolic compounds differently depending on metabolic status. Hence, we evaluated the bioavailability of (poly)­phenols from GSPE in healthy and obese rats.

## 2 Materials and Methods

### 2.1 Grape Seed Proanthocyanidins Extract (GSPE)

GSPE was obtained from white grape seeds and provided by *Les Dérives Résiniques et Terpéniques* (Dax, France). The main phenolic compounds (flavan-3-ols and phenolic acids) present in the extract are listed in Table 1. Nomenclature according to Kay et al.

**Table 1:** Main Phenolic Compounds (Flavan-3-ols and Phenolic Acids) of the Grape Seed Proanthocyanidins Extract Used in This Study, Analysed by HPLC-MS/MS^a^

phenolic compound	concentration (mg/g)
3,4-dihydroxybenzoic acid	1.40 ± 0.25
(+)-catechin	51.88 ± 5.56
(−)-epicatechin	62.86 ± 8.32
3,4,5-trihydroxybenzoic acid	44.66 ± 7.76
kaempferol-3-*O*-glucoside	0.50 ± 0.02
naringenin-7-glucoside	0.64 ± 0.08
*p*-coumaric acid	0.09 ± 0.01
quercetin	0.05 ± 0.01
quercetin-3-*O*-galactoside	0.43 ± 0.05
4-hydroxy-3-methoxybenzoic acid	0.09 ± 0.01
procyanidin dimer	76.84 ± 15.76
procyanidin trimer	13.04 ± 0.64
procyanidin tetramer	5.14 ± 0.28
dimer gallate	15.22 ± 2.72
epicatechin gallate	14.24 ± 2.76
epigallocatechin gallate	0.06 ± 0.01

a Adapted from Rodríguez et al.^21^ Concentrations are expressed as mg of compound per gram of fresh extract (means ± standard deviation).

### 2.2 Chemical and Reagents

The chemical substances and reagents used in the present study have been previously documented by our group.^20^ Acetone, acetonitrile, phosphoric acid (Sigma-Aldrich, Madrid, Spain), glacial acetic acid (Panreac, Barcelona, Spain) and methanol (Scharlab S.L., Barcelona, Spain) were all HPLC analytical quality. Ultrapure water was obtained from a Milli-Q Advantage A10 system (Millipore, Madrid, Spain).

Individual stock standard solutions were prepared at a concentration of 2000 mg/L for various compounds including (+)-catechin, 3,4,5-trihydroxybenzoic acid, 4-hydroxy-3-methoxybenzoic acid, 3-hydroxybenzoic acid, 3′-hydroxyphenylacetic acid, 3′,4′-dihydroxycinnamic acid, 3-(4′-hydroxyphenyl)­propanoic acid, benzoic acid, hippuric acid, 4′-hydroxy-3′-methoxycinnamic acid and benzene-1,2-diol (internal standard; IS) (all purchased from Fluka/Sigma-Aldrich, Madrid, Spain), and proanthocyanidin B2 (Extrasynthese Lyon, France), were prepared using methanol as the solvent and stored in dark glass flasks at −20 °C.

Furthermore, a weekly stock solution containing all the individual compounds was prepared at a concentration of 2000 ppm in methanol. To establish the calibration curve, the standards were combined in a mixture of acetone/water/acetic acid (70/29.5/0.5, v/v/v). This solution was stored in dark glass containers at −20 °C until chromatographic analysis.

### 2.3 Experimental Design

Ninety-six male Fischer 344 rats (F344) at the age of 13 weeks were obtained from Janvier Laboratories (France). Rats were housed in pairs under standard conditions, including a temperature of 22 ± 1 °C, relative humidity of 50–55%, and a 12:12 h light/dark cycle. They had free access to water and a standard chow diet (ST) consisting of 72% carbohydrates, 8% lipids and 19% protein (Safe-A04c, Barcelona, Spain) for 1 week as an acclimation period.

Rats were weighed and randomly assigned to 12 groups (*n* = 8) based on their diet (standard and cafeteria), photoperiod (L12, L6, L18) and treatment (GSPE and vehicle) (see Figure 1). From then on, rats were fed either ST or cafeteria (CAF) diet throughout the entire experimental period. The CAF diet consisted of highly palatable and energy-dense human foods known to induce hyperphagia and obesity^22,23^ and had a composition of 58% carbohydrates, 31% lipids, and 11% protein. The CAF diet was freshly prepared daily and included the following components per rat per day: biscuits with pâté and cheese (15–17 g), bacon (7–10 g), *ensaimada* (pastry) (10–15 g), carrots (11–12 g), standard chow (20–25 g), and milk containing 22% sucrose (w/v). It has previously been shown that the duration of CAF administration used in this study was suitable for the development of metabolic syndrome.^24^ In addition, throughout the experimental procedure, rats were assigned to three different photoperiods for a period of 9 weeks: short photoperiod (L6, 6 h of light and 18 h of darkness), standard photoperiod (L12, 12 h of light and 12 h of darkness), or long photoperiod (L18, 18 h of light and 6 h of darkness). Body weight gain was routinely monitored throughout the experiment to ensure normal growth and health status, following the same procedures described in our previous work using this rat strain and photoperiod model.^25^

**Figure 1:**
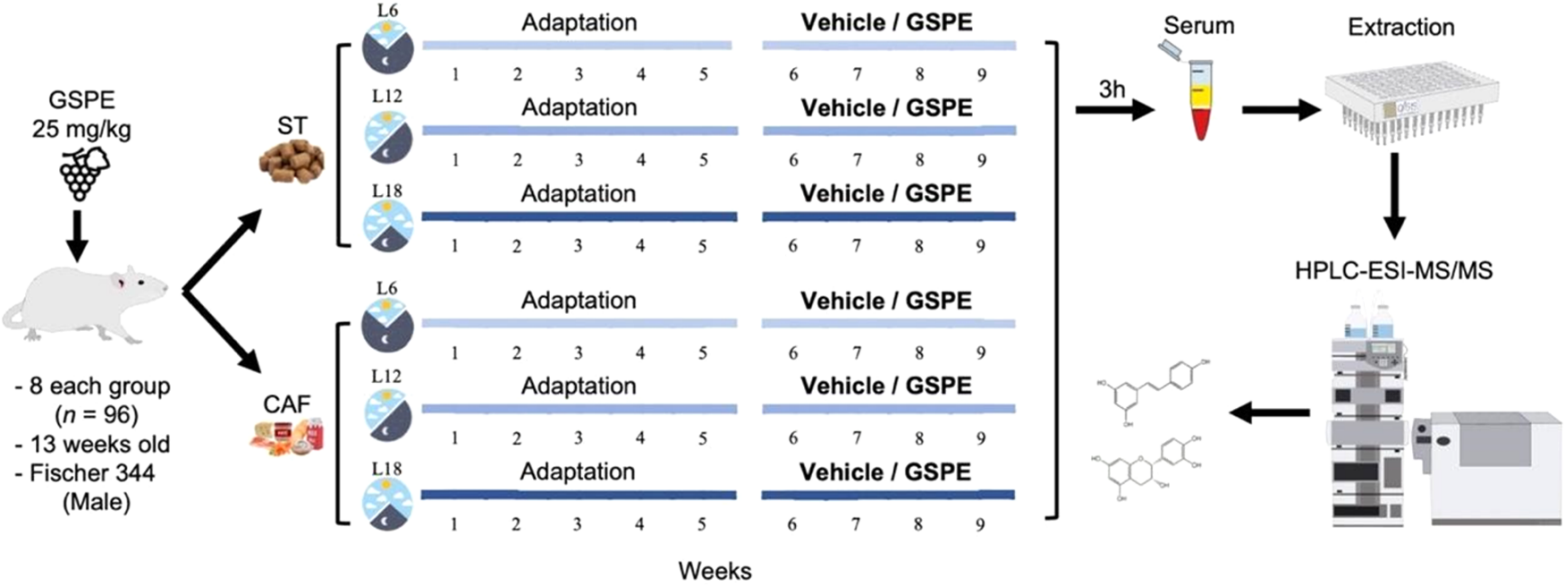
Graphical representation of the experimental design used in this study. 13-weeks-old male Fischer 344 rats were randomly divided into 12 groups (*n* = 8) based on diet (standard or cafeteria), photoperiod (L12, L6, L18) and treatment (GSPE or vehicle). During the last 4 weeks, the GSPE group received a daily oral dose of GSPE (25 mg/kg) diluted in condensed milk and water, while the vehicle group received only the vehicle. Serum samples were collected by decapitation, and the collected blood was centrifuged to obtain serum, which was stored for further analysis. *Abbreviations*: ST, standard chow diet; CAF, cafeteria diet; L6, short photoperiod (6 h light/18 h dark); L12, standard photoperiod (12 h light/12 h dark); L18, long photoperiod (18 h light/6 h dark); GSPE, grape seed proanthocyanidins extract.

### 2.4 Dosage Information/Dosage Regimen

During the last 4 weeks of the experiment, rats were administered a daily oral dose of GSPE equivalent to dietary intake levels (25 mg/kg of body weight) dissolved in condensed milk diluted with water (1/4, v/v), allowing the rats to drink it from the tip of a syringe. A stock solution at 25 mg/mL was prepared so that the volume administered to each rat was adjusted depending of body weight (i.e., 500 μL if body weight was 500 g). This dose has been extensively used by the research group and has been shown to be the lowest and most effective dose in modulating various central metabolic pathways in rats.^26^ Furthermore, considering the translation of animal doses to humans and estimating the daily intake for a 70 kg human,^27^ the dose of 25 mg/kg GSPE corresponds to an intake of approximately 370 mg of phenolics per day. This amount of phenolic compounds can be easily obtained by humans from a diet rich in (poly)­phenols. Control animals received the vehicle only (condensed milk diluted in water, 1/4 v/v). The vehicle and GSPE treatments were administered at 8 a.m. The experimental protocol was originally designed within a broader research project focused on metabolic and genetic responses to chronic GSPE supplementation under different photoperiod conditions. Therefore, serum samples were collected 3 h after the final dose to allow parallel analysis of gene expression and metabolic biomarkers. This time point was selected as a compromise to capture relatively high circulating levels of phenolic metabolites, based on previous pharmacokinetic data indicating peak plasma concentrations between 2 and 3 h after oral administration. All animals were housed under strict photoperiod conditions (L6, L12, or L18) with lights on at 08:00 (ZT0), so the active (dark) period ended at the same time for all groups. Rats were not fasted prior to the last GSPE dose, as the aim was to mimic a realistic chronic intake scenario rather than an acute fasting challenge. To account for possible variability in nocturnal food intake across photoperiods, matched vehicle control groups were included under identical conditions, allowing us to normalize background metabolite levels and minimize dietary interference.

### 2.5 Sacrifice and Serum Collection

After sacrifice by decapitation, serum samples were obtained from the collected blood in nonheparinized tubes. The blood was incubated at room temperature for 1 h and then immediately centrifuged at 1200*g* for 15 min to isolate the serum fraction. The obtained serum samples were subsequently stored at −80 °C until ready for chromatographic analysis, as shown in Figure 1. All procedures performed in this study were approved by the Animal Ethics Committee of the University Rovira i Virgili (Tarragona, Spain) and the Generalitat de Catalunya, in accordance with the EU Directive 2010/63/EU on animal experimentation, under reference number 9495.

### 2.6 Microsolid Phase Serum Phenolic Metabolites Extraction

An internal standard (benzene-1,2-diol) was added to each serum sample prior to extraction at a final concentration equivalent to 50 μL of an internal standard solution at 20 ppm, to ensure consistent recovery and accurate quantification of target metabolites. Before analysis, serum samples were cleaned and concentrated using microsolid phase extraction (μSPE) with 30 μm OASIS HLB μ-Elution Plates (Waters, Barcelona, Spain), following the procedure described in our previous studies.^20^

### 2.7 Chromatographic Analysis (HPLC-ESI-MS/MS)

Chromatographic separation of phenolic compounds in μ-SPE eluted solutions was performed using an Agilent 1290 LC Series and a Zorbax SB-Aq chromatographic column (150 mm × 21 mm i.d., 3.5 μm particle size, Agilent Technologies Palo Alto, CA, USA) at room temperature. The mobile phase consisted of 0.2% acetic acid in water (solvent A) and 100% acetonitrile (solvent B). A specific elution gradient was employed: starting with 5% solvent B, the proportion of solvent B was linearly increased to 55% over a period of 10 min, further increased to 80% B in 2 min, maintained at 80% B for 3 min, and finally returned to 5% solvent B for 1 min. Following elution, a post run of 10 min was applied for column equilibration. The flow rate was set at 0.4 mL/min, and the injection volume for all runs was 2.5 μL. Mass spectrometry analysis was conducted in negative electrospray (ESI) mode at unit resolution. The electrospray capillary voltage was set to 3000 V, the source temperature was maintained at 200 °C and the flow rate was set to 14 L/min with a nebulizer gas pressure of 20 psi. The MS/MS data were acquired in “Multiple Reaction Monitoring” (MRM) mode. Optimized MRM conditions for the analysis were performed as previously reported for the quantification of phase-II and microbial flavan-3-ols metabolites in plasma.^17,20^

### 2.8 Sample Quantification

For sample quantification, a calibration curve was constructed by spiking standard compounds into blank serum from the control group at eight different concentrations ranging from 20 to 5000 ppb. To determine the concentrations of metabolites in the GSPE groups, the concentrations of compounds quantified in the VH group within each photoperiod and diet were subtracted. Sample quantification was achieved by interpolating the analyte/internal standard (IS) peak abundance ratio in the calibration curves. The optimized MRM conditions used in the analysis are given in Table S1 (see Supporting Information). To validate the quantitative method, various parameters including calibration curves, linearity, limit of detection (LOD), limit of quantification (LOQ) and accuracy were assessed (Table S2, see Supporting Information). Standard curves were constructed for each analyte using peak area data and linear least-squares regression was employed to calculate the slope, intercept, and correlation coefficient (*R* ^2^), all of which exhibited *R* ^2^ values exceeding 0.975. The sensitivity of the analytical method was evaluated by determining the LOD, defined as the concentration corresponding to three times the signal-to-noise ratio, and the LOQ, defined as the concentration corresponding to ten times the signal-to-noise ratio. The detection and quantification limits are detailed in Table S2 (see Supporting Information). Data acquisition was performed using MassHunter Software (Agilent Technologies, Palo Alto, CA, USA).

### 2.9 Statistical Analysis

Statistical analyses were conducted using IBM SPSS Statistics version 29.0 (SPSS Inc., Chicago, IL, USA). Differences between groups were examined through one-way and two-way analysis of variance (ANOVA). Initially, the suitability of parametric or nonparametric tests was determined based on the nature of the data. The assessment of normality was conducted using the Shapiro–Wilk test, while Levene’s test was employed to evaluate homoscedasticity across groups. Grubbs test was used to check for outliers at a significance level of α = 0.05. Two-way ANOVA (for parametric analyses) or Krustal–Wallis (for nonparametric analyses) were carried out to evaluate the effects of diet, photoperiod and their interactions. The results were reported in tables and figures with italic capital letters indicating a significant effect of diet (*D*), photoperiod (*L*) or their interaction (*L* × *D*). Following the identification of statistically significant main effects or their combinations, further analyses were performed. For dichotomous variables, T-Student test (parametric) or Mann–Whitney *U* test (nonparametric) were used. For factors with more than two levels, one-way ANOVA or Kruskal–Wallis test followed by multiple comparisons was employed. *Post hoc* contrasts using Bonferroni significant difference (BSD) method were employed when variances between groups were comparable, whereas the Tamhane’s T2 test was used if this assumption was not fulfilled. Results are presented as means with their corresponding standard deviations (SD). Specific statistical tests employed for each analysis are provided in the figure legends. Partial least-squares discriminant analysis (PLS-DA) was performed to evaluate, under multivariate approach, the influence of different factors on the metabolization of phenolic compounds using MetaboAnalyst 5.0 (https://www.metaboanalyst.ca/).

## 3 Results

To evaluate whether administration of GSPE under exposure to different photoperiods affects its bioavailability and metabolism in both healthy and diet-induced obesogenic conditions, the levels of circulating phenolic compounds were analyzed by HPLC-ESI-MS/MS (Table 2). In order to identify differences in concentration due to GSPE consumption, the net increase in phenolic compound concentrations relative to their vehicle or dietary levels was calculated. Specifically, the concentration of metabolites in the vehicle-treated groups was subtracted from that in the GSPE-treated groups. The raw concentration data are presented in Supporting Information (see Tables S3 and S4). Finally, a multivariate analysis was performed to analyze the main effect of photoperiod (*L*), diet (*D*) and their interaction on the serum bioavailability of the main phenolic metabolites (flavan-3-ols, phase-II and microbial colonic metabolites).

**Table 2:** Phenolic Compounds and Their Derivatives Quantified in Serum (μM) 3 h after the Last Acute Dose for 4 Weeks of GSPE (25 mg/kg) by HPLC-ESI-MS/MS[Table-fn t2fn1]

		standard diet (ST)			cafeteria diet (CAF)		
compound	effect	L6	L12	L18	L6	L12	L18
**∑flavan-3-ols**		**0.03 ± 0.031**	**0.04 ± 0.022**	**0.039 ± 0.027**	**0.044 ± 0.044**	**0.018 ± 0.007**	**0.01 ± 0.007**
(+)-catechin	*L* × *D*	0.004 ± 0.004(b)	0.019 ± 0.004(a)	0.006 ± 0.006(b)	0.019 ± 0.018	0.01 ± 0.006	0.01 ± 0.007
(−)-epicatechin		0.03 ± 0.03	0.021 ± 0.02	0.033 ± 0.028	0.025 ± 0.028	0.008 ± 0.007	n.q.
procyanidin dimer B1[Table-fn t2fn2]		n.d.	n.d.	n.d.	n.d.	n.d.	n.d.
procyanidin dimer B2		n.d.	n.d.	n.d.	n.d.	n.d.	n.d.
3,4,5-trihydroxybenzoic acid		n.d.	n.d.	n.d.	n.d.	n.d.	n.d.
4-hydroxy-3-methoxybenzoic acid		n.d.	n.d.	n.d.	n.d.	n.d.	n.d.
**∑phase-II flavan-3-ols**	***D***	**33.256 ± 17.062**	**36.004 ± 11.42**	**29.344 ± 13.696**	**14.729 ± 9.1**	**5.137 ± 1.991**	**4.105 ± 1.969**
(+)-catechin gluc[Table-fn t2fn3]		5.857 ± 4.446	5.917 ± 4.014	6.492 ± 4.275	2.633 ± 2.613	n.d.	n.d.
(−)-epicatechin gluc[Table-fn t2fn4]		9.577 ± 8.81	6.294 ± 4.213	7.17 ± 4.162	5.054 ± 5.233	n.d.	n.d.
methyl-catechin gluc[Table-fn t2fn3]	*D*	0.944 ± 0.792	1.832 ± 0.862	1.021 ± 1.072	0.37 ± 0.148	0.242 ± 0.196	0.112 ± 0.116
methyl-epicatechin gluc[Table-fn t2fn4]	*D*	15.974 ± 5.819	20.978 ± 6.327	13.881 ± 7.222	6.32 ± 1.71	4.648 ± 1.696	3.804 ± 1.839
catechin sulfate[Table-fn t2fn3]		n.d.	n.d.	n.d.	n.d.	n.d.	n.d.
epicatechin sulfate[Table-fn t2fn4]		n.d.	n.d.	n.d.	n.d.	n.d.	n.d.
3-*O*-methylgallic acid[Table-fn t2fn5]	*D*	0.034 ± 0.019	0.057 ± 0.029	0.049 ± 0.036	0.037 ± 0.023	0.034 ± 0.026	0.016 ± 0.007
3-*O-*methyl epicatechin[Table-fn t2fn4]		n.d.	n.d.	n.d.	n.d.	n.d.	n.d.
4-*O-*methyl epicatechin[Table-fn t2fn4]		n.d.	n.d.	n.d.	n.d.	n.d.	n.d.
methyl-cate/epi sulfate[Table-fn t2fn3],[Table-fn t2fn4]	*D*	0.869 ± 0.499	0.925 ± 0.081	0.731 ± 0.453	0.314 ± 0.16	0.214 ± 0.098	0.173 ± 0.104
**∑microbial metabolism**		**<VL**	**13.002 ± 15.559**	**<VL**	**10.895 ± 21.437**	**5.775 ± 16.349**	**<VL**
phenylacetic acid[Table-fn t2fn6]	*D*, *L* × *D*	<VL	<VL	<VL	<VL	0.267 ± 1.263	0.054 ± 1.657([Table-fn t2fn9])
3-(4′-hydroxyphenyl) propanoic acid	*L*, *L* × *D*	0.023 ± 0.11	<VL	<VL	0.121 ± 0.429(A)	<VL (AB)	0.087 ± 0.172(A)
3′,4′-dihydroxyphenylacetic acid		n.d.	n.d.	n.d.	n.d.	n.d.	n.d.
3′-hydroxyphenylacetic acid	*D*, *L*, *L* × *D*	<VL	<VL	<VL	<VL	0.473 ± 0.432([Table-fn t2fn9])	<VL
4′-hydroxyphenylacetic acid		n.d.	n.d.	n.d.	n.d.	n.d.	n.d.
4′-hydroxy-3′-methoxyphenylacetic acid[Table-fn t2fn7]	L × *D*	<VL	6.621 ± 8.388	<VL	17.143 ± 19.256([Table-fn t2fn9]A)	0.247 ± 9.192(AB)	<VL(B)
hippuric acid		<VL	3.257 ± 5.288	<VL	<VL	3.035 ± 7.242	1.136 ± 5.372
3′,4′-dihydroxycinnamic acid		<VL	3.262 ± 3.791	<VL	<VL	2.311 ± 5.697	<VL
4′-hydroxy-3′-methoxycinnamic acid	*D*, *L*, *L* × *D*	<VL	0.001 ± 0.021	<VL	<VL(B)	<VL(AB)	0.044 ± 0.059(A)
benzoic acid		<VL	0.067 ± 0.335	<VL	<VL	<VL	<VL
3-hydroxybenzoic acid		n.q.	n.q.	n.q.	n.q.	n.q.	n.q.
phenylpropionic acid[Table-fn t2fn8]		<VL	1.102 ± 1.498	<VL	n.d.	n.d.	n.d.

1 Abbreviations: VL, vehicle level; L6, short photoperiod (6 h light/18 h dark); L12, standard photoperiod (12 h light/12 h dark); L18, long photoperiod (18 h light/6 h dark); not detected (n.d.); not quantified (n.q.); glucuronide (gluc).

2 Quantified using the calibration curve of procyanidin dimer B2.

3 Quantified using the calibration curve of catechin.

4 Quantified using the calibration curve of epicatechin.

5 Quantified using the calibration curve of 3,4,5-trihydroxybenzoic acid.

6 Quantified using the calibration curve of 3′-hydroxyphenylacetic acid.

7 Quantified using the calibration curve of 4-hydroxy-3-methoxybenzoic acid.

8 Quantified using the calibration curve of 3-(4′-hydroxyphenyl) propanoic acid. The entire statistical procedure is described in the Section 2.9. “Effect” column represents the statistical results (*p* < 0.05) of diet (*D*), photoperiod (*L*) or their interaction (*L* × *D*). When interaction was also significant, BSD or Tamhane’s T2 *post hoc* test was performed. When comparing the photoperiods in each diet, lowercase letters (a, b, c) indicate significant differences in photoperiods for rats fed the ST diet and uppercase letters (A, B, C) for rats fed the CAF diet. Same letters indicate no significant difference; different letters indicate statistically significant differences.

9 Indicates significant variance between the different diets for the same photoperiod. Where there was no statistically significant difference between groups, the letters are not shown. Results are expressed as μM ± SD (*n* = 8). The significance level was *p* < 0.05. Note: negative net differences were interpreted as no measurable increment over the vehicle group and are indicated as “<vehicle”.

First, partial least-squares discriminant analysis (PLS-DA) was employed as the primary analytical approach, aiding in the identification of differences in total (poly)­phenols bioavailability across the treatment groups. The PLS-DA score plot revealed that samples clustered differently depending on the diet, indicating that (poly)­phenols bioavailability is different in rats feed standard diet compared to rats feed CAF diet (Figure 2A). In contrast, differences were less evident for the photoperiod factor (Figure 2B), although it displayed a lower level of data dispersion under L12 conditions in comparison to L6 or L18. To examine the primary contributors to the separation observed in the PLS-DA components, we compared the metabolic loadings within the Variable Importance in Projection (VIP) for the respective treatments. Figure 2C,D present the top 15 compounds that significantly contributed to separate the treatments.

**Figure 2:**
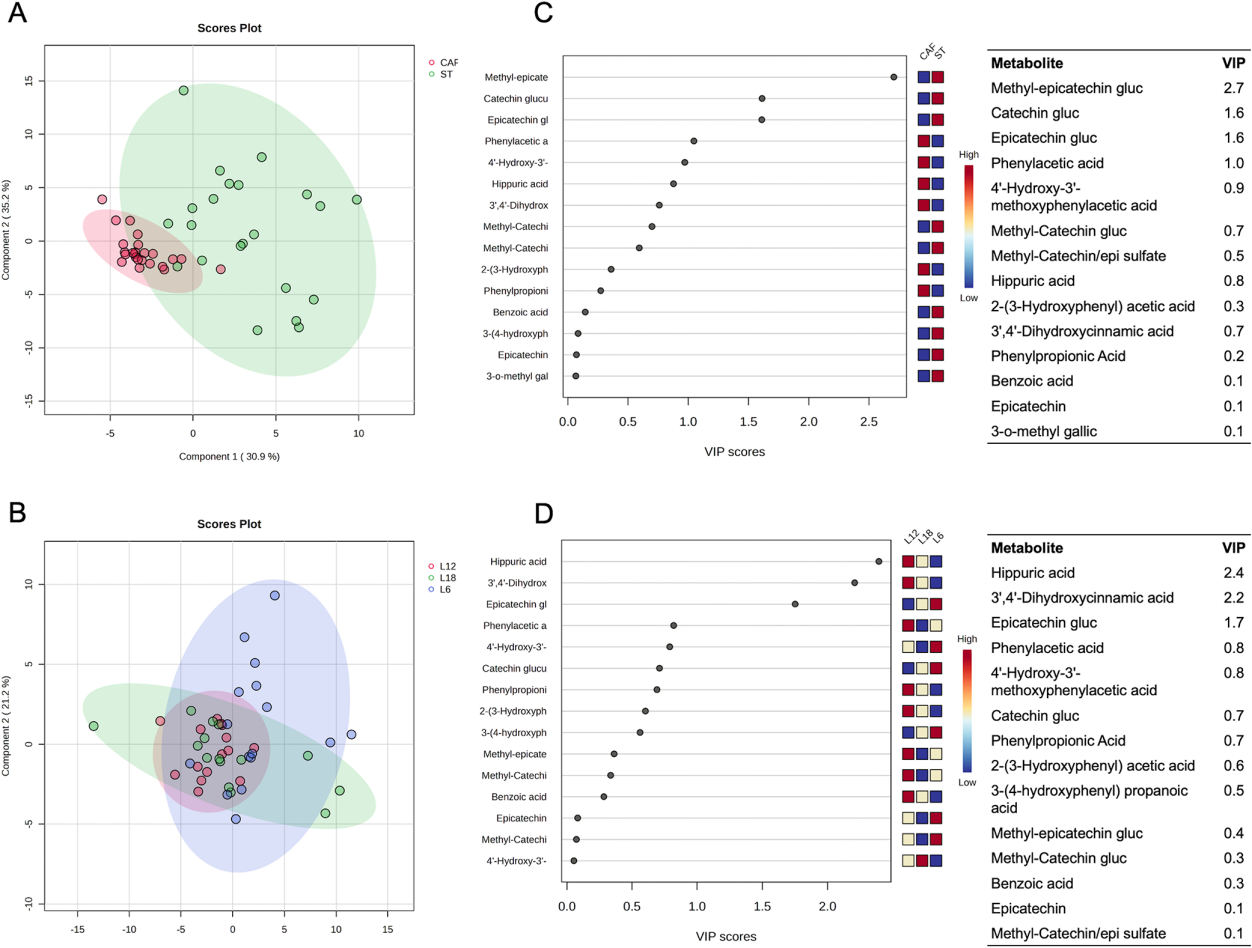
Score plots derived from Partial Least Squares Discriminant Analysis (PLS-DA) (A, B) and Variable of Importance (VIP) scores (C, D). In panel (A), the color coding distinguishes between diet treatments, with red dots representing the CAF group and green dots signifying the ST group. The *X*-axis and *Y*-axis are labeled as the first and second principal components, accounting for 30.9% and 35.2% of the total variation, respectively. Plot (B), corresponds to photoperiod treatment, where red, green, and blue dots correspond to L12, L18, and L6, respectively. Component 1 accounts for 39.9% of the total variation, while Component 2 contributes 21.2%. Tables on the right report the highest VIP values for Component 2 (C) and Component 1 (D).

### 3.1 Total (Poly)­phenolic Compounds

Figure 3A illustrates the total content of identified and quantified (poly)­phenolic compounds. Differences in photoperiod effects were observed depending on the diet. Interestingly, rats fed the ST and housed under L12 conditions had higher levels of metabolites than those housed under L6 and L18 conditions. This photoperiod effect in ST-fed rats was primarily driven by a group of microbiota-derived metabolites that only exceeded vehicle levels in L12 conditions. Conversely, this pattern was not observed in the CAF group. Actually, the concentration levels of phenolic compounds in CAF-fed rats housed under L12 conditions were significantly lower than those fed the ST. Additionally, a trend was observed in rats fed with CAF and housed in L6 conditions, as they had higher metabolite levels than those housed in L12 and L18 conditions, which is consistent with our previous study.^17^ Finally, among the different groups of metabolites listed in Table 2, phase II-derived metabolites were the most abundant, followed by microbiota-derived metabolites. Flavan-3-ols were the least abundant.

**Figure 3:**
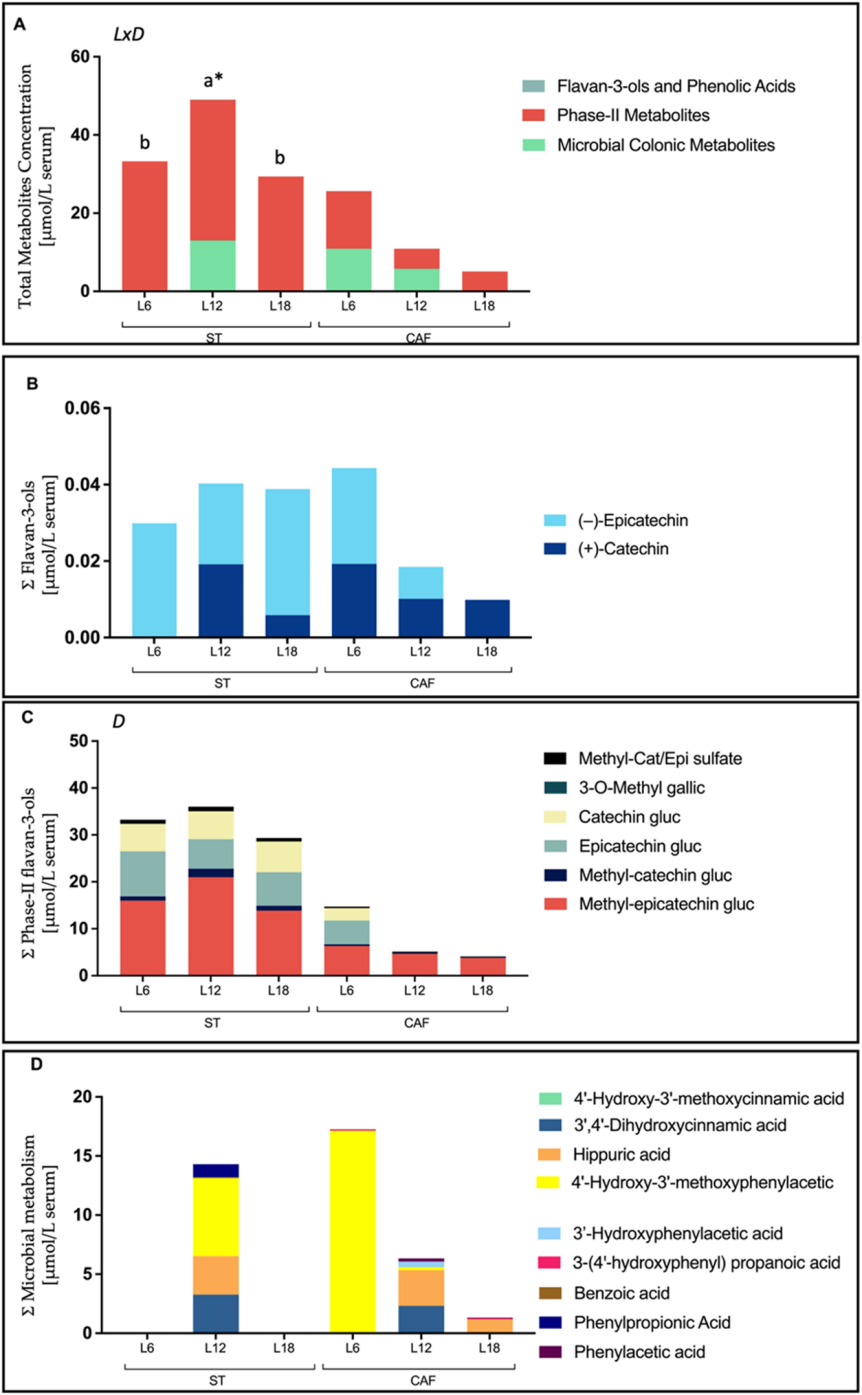
Distribution of quantified phenolic compounds and their derivatives in serum 3 h after the last dose of GSPE (25 mg/kg) (4 weeks) analyzed by HPLC-ESI-MS/MS and with VH levels subtracted. The figure shows the levels of total (poly)­phenolic compounds (A), flavan-3-ols (B), phase II flavan-3-ols metabolites (C), and microbial colonic metabolites (D). Rats were divided into 12 groups (*n* = 8), according to diet (ST and CAF), photoperiod (L12, L6, L18) and treatment (GSPE or vehicle). Statistical comparisons between the groups were conducted using two- and one-way ANOVA. Graphs show the statistical effect (*p* < 0.05) for diet (D), photoperiod (L), and their interaction (LxD). Where one-way ANOVA was significant, post hoc tests (BSD or Tamhane’s T2) were performed to determine differences between the means. Lowercase letters (a, b, c) represent significant differences in photoperiods for rats fed the ST diet* Indicates significant differences between the different diets for the same photoperiod. Abbreviations used are L6 for short photoperiod (6 h of light/18 h of darkness), L12 for standard photoperiod (12 h of light/12 h of darkness), L18 for long photoperiod (18 h of light/6 h of darkness), and glucuronide (gluc).

### 3.2 Flavan-3-ols

Figure 3B and Table 2 show the quantification and identification of different GSPE nonmetabolized metabolites (group of flavan-3-ols) present in serum. Within this group, there was no apparent effect or rhythmicity due to photoperiod variations in rats fed with ST. However, a significant difference was observed in (+)-catechin levels, with higher concentrations in L12 compared to L6 and L18 among rats on the standard diet. In contrast, although it did not reach statistical significance, a trend toward higher concentration of these metabolites was observed in rats fed a CAF diet under L6 conditions.

Additionally, the correlation heatmap showed a positive correlation (coefficient >0.5) between phase-II derived metabolites and epicatechin. These findings highlight the importance of (−)-epicatechin in the metabolic profile (Figure S1A, see Supporting Information).

### 3.3 Phase-II Flavan-3-ols Metabolites

Figure 3C and Table 2 show the metabolites identified and quantified from phase-II metabolism. This group comprises the highest concentration of bioavailable metabolites in serum. The compounds that significantly contributed the most were methyl-epicatechin glucuronide (2.7 VIP score), (−)-epicatechin glucuronide (1.6 VIP score) and (+)-catechin glucuronide (1.6 VIP score). This large group is clearly influenced by dietary factors, exhibiting significantly higher bioavailability in rats fed the ST diet compared to the CAF diet (*p* < 0.001). Similarly, pattern hunter analysis (Figure S1B, see Supporting Information) identified a positive correlation (>0.5) among ST consumption and serum bioavailability of these metabolites.

### 3.4 Microbial Colonic Metabolites

Finally, the bioavailability of microbiota-derived compounds, which is the second-largest group identified in the samples, is shown in Figure 3D and Table 2. The behavior of this group is highly diverse, suggesting a complex interplay of factors influencing their production. After subtracting the vehicle concentrations used as a blank, we identified groups of rats that had concentrations below vehicle levels, suggesting that both photoperiod and dietary stress cause a change in the metabolic environment of the microbiota. Within the ST-fed group, the L12 photoperiod was found to be the most favorable to produce microbiota-derived metabolites, consistently exceeding vehicle levels. In contrast, the concentrations were below the vehicle level in ST-fed rats housed under L6 or L18 conditions. However, rats on the cafeteria diet showed concentrations above vehicle levels, although each photoperiod exhibited different serum metabolite profiles. In particular, CAF-fed rats housed under L12 conditions showed a similar pattern to that observed in ST diet-fed rats but with a significant reduction in the concentration of these metabolites. Thus, compounds such as 4′-hydroxy-3′-methoxyphenylacetic acid, was significantly higher under L6 photoperiod compared to any of the other groups. All other differences belonging to the concentration of the individual microbiota-derived compounds are shown in Table 2.

## 4 Discussion

Our results support the role of (poly)­phenols in modulating cardiovascular and metabolic pathways, in the line with previous findings on their health benefits.^1^ However, to fully understand these benefits, it is crucial to investigate their bioavailability. Various factors, including compound structure, environmental conditions, sex, age, gut microbiota and diet have been shown to affect how these compounds are absorbed and metabolized.^12,14–16,18^ Biological rhythms, including circadian and seasonal rhythms, are emerging as important factors affecting the bioactivity of (poly)­phenols.^19^ Annual cycles, known as circannual or seasonal rhythms, are influenced by the Earth’s movement around the sun, resulting in seasonal changes.^28^ In this context, exposure to different photoperiods (the number of light hours per day) significantly influences the behavior, physiology, and metabolism of mammals, leading to changes in physical activity, energy expenditure, and body fat, particularly in regions with pronounced seasons.^29,30^ Disruptions in these rhythms are linked to disease development, affecting physiological aspects like plasma estradiol levels, kidney glomerular filtration rate, metabolic rate, and gene expression governing physiological processes, thus potentially influencing (poly)­phenol bioavailability.^31^ Additionally, fluctuations in light/dark patterns are associated with metabolic disturbances, contributing to the onset of metabolic syndrome.^32,33^ This exposure to varied photoperiods has demonstrated effects on serum lipid levels, insulin sensitivity, body mass, and adiposity in both human^34^ and animal studies.^35–37^

Hence, the present study aimed to evaluate whether circannual rhythms affect serum bioavailability of phenolic compounds differently depending on health conditions. Fischer 344 rats were selected because they are characterized by a high sensitivity to biological rhythms.^18^ According to Togo et al., this metabolic response to daylength is evident in this strain which show a particular adaptability to photoperiod variations.^38^ Furthermore, our recent findings indicate an effect on bioavailability due to the timing of dose administration in Fischer 344.^20^

In this chronic study, we analyzed the main families of (poly)­phenols and their metabolized derivatives, including flavan-3-ols, phase II derivatives and microbial colonic metabolites at 3 h after the last GSPE administration. This time point was selected to align with previous pharmacokinetic studies indicating that peak plasma concentrations of phase II flavan-3-ol metabolites occur within the first 2–3 h postingestion. However, a limitation of this study is the use of a single time-point sampling, which does not allow for a complete pharmacokinetic characterization, particularly for microbial-derived metabolites that reach peak concentration later. Future studies with multiple sampling points would be necessary to confirm absorption kinetics more precisely and to better elucidate the underlying circadian mechanisms that may explain the photoperiod effect. Xenobiotic-metabolizing pathways including phase I (cytochromes P450), phase II (e.g., UDP-glucuronosyltransferases), and phase III transporters are subject to daily oscillations influenced by clock genes and feeding behavior, as described in studies of seasonal and circadian regulation.^28,29^ Additionally, gut physiology and microbiota composition have been shown to fluctuate in relation to light/dark cycles and feeding patterns, affecting nutrient processing and host metabolic responses.^19,31,32^ Although these mechanisms were not directly measured in our study, they represent plausible biological pathways by which photoperiod may shape (poly)­phenol absorption and metabolism. Future investigations should test these hypotheses explicitly under varying photoperiods.

In our data set, phase II metabolites were the most abundant, followed by microbiota-derived metabolites, with flavan-3-ols being the least abundant. This aligns with our previous studies suggesting that the highest levels of flavan-3-ols and their phase II metabolites are achieved during the initial hours after ingestion.^20,39,40^ At the same time, microbial metabolites reach their peak concentration between 7 to 24 h after consumption and can remain in the blood for up to 48 h in some cases.^41^

A multivariate approach was employed to assess the combined influence of diet and photoperiods on the bioavailability of phenolic compounds derived from GSPE consumption. This chronic study demonstrates how metabolic disruption resulting from the diet contributes to the onset of metabolic syndrome. In the CAF diet-fed rat group, a unification of diversity and a reduction in the bioavailability of total metabolites, flavan-3-ols, and transformed by phase II, were observed. This phenomenon was not observed in the ST diet-fed rat groups, where greater bioavailability and variability of phenolic compounds were found. This discrepancy may be attributed to the absence of a unifying element such as the alteration caused by the diet. In contrast, the difference in photoperiod, regardless of diet, did not result in a clear separation between groups in the PLS-DA multivariate analysis.

The interaction of both factors (diet and photoperiod) on the bioavailability of total metabolites was analyzed, concluding that within a “standard” health state (ST), differences in light exposure hours significantly affected the bioavailability of phenolic compounds. Hence, rats fed a standard diet and exposed to a 12 h photoperiod (L12) exhibited higher levels of total metabolites compared to those under shorter (L6) or longer (L18) photoperiods. This apparent higher bioavailability in SD-fed rats under L12 conditions, despite their nocturnal behavior, may reflect additional factors not fully captured in this study, such as lower metabolic stress compared to CAF-fed animals or interactions with feeding patterns during light exposure.

However, CAF group did not exhibit the same pattern. The cafeteria diet, comprising highly palatable and energy-dense foods, appears to alter the relationship between photoperiod and metabolite levels.^23^ Thus, in a state of obesity and metabolic disturbance, there is an initial alteration resulting in lower bioavailability, translating to a reduced influence of photoperiods. In the same way as in our previous work, rats fed a CAF diet showed higher bioavailability and variability of total phenolic compounds under the shorter photoperiod or, equivalently, a longer duration of the active phase (L6), compared to other photoperiods (L12, L18).^17^ This finding aligns with Larkin et al. work, which demonstrated that rats consume most of their daily food intake during the dark phase.^42^ We believe that a longer active phase leads to increased food consumption and, consequently, greater bioavailability of (poly)­phenols from the diet. This aligns with recent results from Soliz-Rueda et al., reporting differences in energy intake in CAF-fed rats depending on the photoperiod, with a tendency to increase food and energy intake under short light conditions.^43^ Notably, this difference in energy intake was mainly attributed to increased carbohydrate intake, possibly linked to higher insulin levels observed in CAF-fed rats under L6 conditions.^44^

However, there is a discrepancy in the literature on this topic. Hence, previous studies have linked short photoperiods to alterations in fat content in rodents.^38,45,46^ Gibert-Ramos et al. evaluated the role of photoperiod on the metabolism of WAT and BAT in Fischer 344 rats and did not observe differences in body weight, only noting a trend of reduced fat in rats fed with an ST on the short photoperiod.^35^ This discrepancy persists in the literature, where uncertainties remain regarding the impact of photoperiod on body weight and energy intake. Some studies suggest that changes in body mass may be linked to fluctuations in food intake,^38,47^ while others, note variations in fat composition without parallel modifications in food consumption.^46^ Shoemaker et al. suggested a complex relationship where decreases in food intake may follow reductions in body weight.^48^

Concerning flavan-3-ols, our data revealed a significant photoperiod-dependent trend in cafeteria diet-fed rats, with the highest concentration found in the L6 photoperiod. This emphasizes the influence of photoperiod in conjunction with dietary stress on the production of flavan-3-ols. However, this pattern was not evident in rats fed a standard diet, indicating a complex interplay between diet, photoperiod, and the metabolite profiles of these compounds. Interestingly, (+)-catechin levels exhibited significant differences across photoperiods in rats on a standard diet, with higher concentrations in the L12 group. On the other hand, phase-II-derived metabolites constituted the most abundant group among the analyzed compounds, and their levels were significantly influenced by dietary factors, with higher bioavailability observed in rats fed a standard diet compared to those fed a cafeteria diet. The dominance of these metabolites underscores the importance of metabolic pathways involving glucuronidation and other phase-II processes in the bioavailability of phenolic compounds. Notably, we identified a positive correlation between (−)-epicatechin and phase-II metabolites, emphasizing the central role of this compound in the metabolic profile. Additionally, similar to the flavan-3-ols group, although variations in photoperiod did not produce significant differences in this group, the metabolite bioavailability levels of the rats exposed under L6 conditions and fed CAF seem to stand out from the other photoperiods. These results are consistent with our previous work.^17^ Similarly, Iglesias-Carres et al. also showed that the bioavailability of grape seed was higher in rats exposed to L6 conditions compared to rats under L18 conditions.^49^ This finding reinforces the theory that the diurnal rhythmicity of phase II enzymes could influence the bioavailability and metabolism of these phenolic compounds, with greater activity during the dark phase when rats are active.

The study found that microbial colonic metabolites exhibited notably diverse behavior, with evidence of complex interactions between photoperiod and diet.^20^ Interestingly, concentrations of these metabolites were below vehicle levels in several rat groups, suggesting that both photoperiod and dietary stress can alter the metabolic environment of the microbiota.^23^ In standard diet-fed rats, the L12 photoperiod appeared to promote the production of microbiota-derived metabolites, consistently exceeding vehicle levels. In contrast, rats on a cafeteria diet showed concentrations above vehicle levels, but with different serum metabolite profiles for each photoperiod. The higher serum phenolic concentrations observed in L6 may be linked to modifications in appetite and feeding patterns driven by photoperiod. Previous studies in Fischer 344 rats show that both L6 and L18 groups display altered caloric intake and patterns of ingestion compared to L12.^50^

In conclusion, this study emphasizes how the influence of circannual rhythms on the bioavailability of (poly)­phenols varies between different dietary health states. A pronounced effect on bioavailability levels was revealed in rats exposed to a 12 h photoperiod and fed with a standard diet. However, this pattern was altered in rats fed with cafeteria diet, suggesting an attenuated influence of photoperiod under obesogenic conditions. These findings contribute to a better understanding of the complex relationships between diet, photoperiod, and serum metabolites.

## Supplementary Material



## Notes

Optimized MRM transitions for detection of phenolic metabolites in rat serum (Table S1); method validation parameters (calibration, LOD, LOQ) (Table S2); phenolic metabolite concentrations in vehicle- and GSPE-treated rats (Tables S3 and S4), and correlation analysis between metabolite profiles, diet, and photoperiod (Figure S1) (PDF)

## ∥

Disease Biomarkers and Molecular Mechanisms Group (DIBIOMEC), Institut d’Investigació Sanitària Pere Virgili (IISPV), C/Dr. Mallafré Guash, 4, 43005 Tarragona, Spain

## References

[1] Del
Rio D., Rodriguez-Mateos A., Spencer J. P. E., Tognolini M., Borges G., Crozier A. (2013). Dietary (Poly)­Phenolics in Human
Health: Structures, Bioavailability, and Evidence of Protective Effects
against Chronic Diseases. Antioxid. Redox Signaling.

[2] Rana A., Samtiya M., Dhewa T., Mishra V., Aluko R. E. (2022). Health
Benefits of Polyphenols: A Concise Review. J.
Food Biochem..

[3] Durazzo A., Lucarini M., Souto E. B., Cicala C., Caiazzo E., Izzo A. A., Novellino E., Santini A. (2019). Polyphenols: A Concise
Overview on the Chemistry, Occurrence, and Human Health. Phytother. Res..

[4] Shen N., Wang T., Gan Q., Liu S., Wang L., Jin B. (2022). Plant Flavonoids: Classification,
Distribution, Biosynthesis, and
Antioxidant Activity. Food Chem..

[5] Chang Y. C., Yang M. Y., Chen S. C., Wang C. J. (2016). Mulberry Leaf Polyphenol
Extract Improves Obesity by Inducing Adipocyte Apoptosis and Inhibiting
Preadipocyte Differentiation and Hepatic Lipogenesis. J. Funct. Foods.

[6] Ding S., Xu S., Fang J., Jiang H. (2020). The Protective
Effect of Polyphenols
for Colorectal Cancer. Front. Immunol..

[7] Shahwan M., Alhumaydhi F., Ashraf G. M., Hasan P. M. Z., Shamsi A. (2022). Role of Polyphenols
in Combating Type 2 Diabetes and Insulin Resistance. Int. J. Biol. Macromol..

[8] Torres-Fuentes C., Suárez M., Aragonès G., Mulero M., Ávila-Román J., Arola-Arnal A., Salvadó M. J., Arola L., Bravo F. I., Muguerza B. (2022). Cardioprotective Properties of Phenolic Compounds:
A Role for Biological Rhythms. Mol. Nutr. Food
Res..

[9] Angelino D., Cossu M., Marti A., Zanoletti M., Chiavaroli L., Brighenti F., Del Rio D., Martini D. (2017). Bioaccessibility
and Bioavailability of Phenolic Compounds in Bread: A Review. Food Funct..

[10] Fernández-García E., Carvajal-Lérida I., Pérez-Gálvez A. (2009). In Vitro Bioaccessibility
Assessment as a Prediction Tool of Nutritional Efficiency. Nutr. Res..

[11] Xiao J. (2017). Dietary Flavonoid
Aglycones and Their Glycosides: Which Show Better Biological Significance?. Crit. Rev. Food Sci. Nutr..

[12] Ozdal T., Sela D. A., Xiao J., Boyacioglu D., Chen F., Capanoglu E. (2016). The Reciprocal Interactions between
Polyphenols and Gut Microbiota and Effects on Bioaccessibility. Nutrients.

[13] Bohn T. (2014). Dietary Factors
Affecting Polyphenol Bioavailability. Nutr.
Rev..

[14] D’Archivio M., Filesi C., Varì R., Scazzocchio B., Masella R. (2010). Bioavailability of the Polyphenols: Status and Controversies. Int. J. Mol. Sci..

[15] Margalef M., Pons Z., Iglesias-Carres L., Arola L., Muguerza B., Arola-Arnal A. (2016). Gender-Related
Similarities and Differences in the
Body Distribution of Grape Seed Flavanols in Rats. Mol. Nutr. Food Res..

[16] Margalef M., Iglesias-Carres L., Pons Z., Bravo F. I., Muguerza B., Arola-Arnal A. (2016). Age Related
Differences in the Plasma Kinetics of Flavanols
in Rats. J. Nutr. Biochem..

[17] Arreaza-Gil V., Escobar-Martínez I., Mulero M., Muguerza B., Suárez M., Arola-Arnal A., Torres-Fuentes C. (2023). Gut Microbiota
Influences the Photoperiod Effects on Proanthocyanidins Bioavailability
in Diet-Induced Obese Rats. Mol. Nutr. Food
Res..

[18] Carere, C. ; Maestripieri, D. Animal Personalities; University of Chicago Press, 2013 10.7208/chicago/9780226922065.001.0001.

[19] Arola-Arnal A., Cruz-Carrión Á., Torres-Fuentes C., Ávila-Román J., Aragonès G., Mulero M., Bravo F. I., Muguerza B., Arola L., Suárez M. (2019). Chrononutrition and Polyphenols: Roles and Diseases. Nutrients.

[20] Escobar-Martínez I., Arreaza-Gil V., Muguerza B., Arola-Arnal A., Bravo F. I., Torres-Fuentes C., Suárez M. (2022). Administration
Time Significantly Affects Plasma Bioavailability of Grape Seed Proanthocyanidins
Extract in Healthy and Obese Fischer 344 Rats. Mol. Nutr. Food Res..

[21] Rodríguez R. M., de Assis L. V. M., Calvo E., Colom-Pellicer M., Quesada-Vázquez S., Cruz-Carrión Á., Escoté X., Oster H., Aragonès G., Mulero M. (2024). Grape-Seed Proanthocyanidin Extract (GSPE) Modulates
Diurnal Rhythms of Hepatic Metabolic Genes and Metabolites, and Reduces
Lipid Deposition in Cafeteria-Fed Rats in a Time-of-Day-Dependent
Manner. Mol. Nutr. Food Res..

[22] Lalanza J. F., Snoeren E. M. S. (2021). The Cafeteria
Diet: A Standardized Protocol and Its
Effects on Behavior. Neurosci. Biobehav. Rev..

[23] Sampey B. P., Vanhoose A. M., Winfield H. M., Freemerman A. J., Muehlbauer M. J., Fueger P. T., Newgard C. B., Makowski L. (2011). Cafeteria
Diet Is a Robust Model of Human Metabolic Syndrome with Liver and
Adipose Inflammation: Comparison to High-Fat Diet. Obesity.

[24] Mas-Capdevila A., Iglesias-Carres L., Arola-Arnal A., Suárez M., Bravo F. I., Muguerza B. (2020). Changes in
Arterial Blood Pressure
Caused by Long-Term Administration of Grape Seed Proanthocyanidins
in Rats with Established Hypertension. Food
Funct..

[25] Rodríguez R. M., Colom-Pellicer M., Hernández-Baixauli J., Calvo E., Suárez M., Arola-Arnal A., Torres-Fuentes C., Aragonès G., Mulero M. (2024). Grape Seed Proanthocyanidin Extract
Attenuates Cafeteria-Diet-Induced Liver Metabolic Disturbances in
Rats: Influence of Photoperiod. Int. J. Mol.
Sci..

[26] Aragonès G., Suárez M., Ardid-Ruiz A., Vinaixa M., Rodríguez M. A., Correig X., Arola L., Bladé C. (2016). Dietary Proanthocyanidins
Boost Hepatic NAD + Metabolism and SIRT1 Expression and Activity in
a Dose-Dependent Manner in Healthy Rats. Sci.
Rep.

[27] Reagan-Shaw S., Nihal M., Ahmad N. (2008). Dose Translation
from Animal to Human
Studies Revisited. FASEB J..

[28] Oike H., Oishi K., Kobori M. (2014). Nutrients,
Clock Genes, and Chrononutrition. Curr. Nutr.
Rep..

[29] Korf H. W. (2018). Signaling
Pathways to and from the Hypophysial Pars Tuberalis, an Important
Center for the Control of Seasonal Rhythms. Gen. Comp. Endrocrinol..

[30] Prendergast B. J. (2005). Internalization
of Seasonal Time. Horm. Behav..

[31] Liu J., Song Y., Lu X., Chen T., Guo W., Fan Z., Kang X., Wang Y., Wang Y. (2018). Seasonal Variation
Influences on Intestinal Microbiota in Rats. Curr. Microbiol..

[32] Coskun A., Zarepour A., Zarrabi A. (2023). Physiological
Rhythms and Biological
Variation of Biomolecules: The Road to Personalized Laboratory Medicine. Int. J. Mol. Sci..

[33] Zimmet P., Alberti K. G. M. M., Stern N., Bilu C., El-Osta A., Einat H., Kronfeld-Schor N. (2019). The Circadian Syndrome: Is the Metabolic
Syndrome and Much More!. J. Intern. Med..

[34] Yoshimura E., Tajiri E., Hatamoto Y., Tanaka S. (2020). Changes in Season Affect
Body Weight, Physical Activity, Food Intake, and Sleep in Female College
Students: A Preliminary Study. Int. J. Environ.
Res. Public Health.

[35] Gibert-Ramos A., Ibars M., Salvadó M. J., Crescenti A. (2019). Response to
the Photoperiod in the White and Brown Adipose Tissues of Fischer
344 Rats Fed a Standard or Cafeteria Diet. J.
Nutr. Biochem..

[36] Navarro-Masip È., Manocchio F., Colom-Pellicer M., Escoté X., Iglesias-Carres L., Calvo E., Bravo F. I., Muguerza B., Desjardins Y., Aragonès G. (2023). Vitis Vinifera
L. Bioactive Components
Modulate Adipose Tissue Metabolic Markers of Healthy Rats in a Photoperiod-Dependent
Manner. Mol. Nutr. Food Res..

[37] Xie X., Zhao B., Huang L., Shen Q., Ma L., Chen Y., Wu T., Fu Z. (2017). Effects of Altered
Photoperiod on Circadian Clock and Lipid Metabolism in Rats. Chronobiol. Int..

[38] Togo Y., Otsuka T., Goto M., Furuse M., Yasuo S. (2012). Photoperiod
Regulates Dietary Preferences and Energy Metabolism in Young Developing
Fischer 344 Rats but Not in Same-Age Wistar Rats. Am. J. Physiol.: Endocrinol. Metab..

[39] Margalef M., Pons Z., Iglesias-Carres L., Bravo F. I., Muguerza B., Arola-Arnal A. (2017). Flavanol Plasma
Bioavailability Is Affected by Metabolic
Syndrome in Rats. Food Chem..

[40] Monagas M., Urpi-Sarda M., Sánchez-Patán F., Llorach R., Garrido I., Gómez-Cordovés C., Andres-Lacueva C., Bartolomé B. (2010). Insights into the Metabolism and
Microbial Biotransformation of Dietary Flavan-3-Ols and the Bioactivity
of Their Metabolites. Food Funct..

[41] Margalef M., Pons Z., Bravo F. I., Muguerza B., Arola-Arnal A. (2015). Plasma Kinetics
and Microbial Biotransformation of Grape Seed Flavanols in Rats. J. Funct. Foods.

[42] Larkin L. M., Moore B. J., Stern J. S., Horwitz B. A. (1991). Effect
of Photoperiod
on Body Weight and Food Intake of Obese and Lean Zucker Rats. Life Sci..

[43] Soliz-Rueda J. R., López-Fernández-Sobrino R., Bravo F. I., Aragonès G., Suarez M., Muguerza B. (2022). Grape Seed
Proanthocyanidins Mitigate
the Disturbances Caused by an Abrupt Photoperiod Change in Healthy
and Obese Rats. Nutrients.

[44] Soliz-Rueda J. R., López-Fernández-Sobrino R., Torres-Fuentes C., Bravo F. I., Suárez M., Mulero M., Muguerza B. (2023). Metabolism
Disturbance by Light/Dark Cycle Switching Depends on the Rat Health
Status: The Role of Grape Seed Flavanols. Food
Funct..

[45] Bartness T. J. (1996). Photoperiod,
Sex, Gonadal Steroids, and Housing Density Affect Body Fat in Hamsters. Physiol. Behav..

[46] Kooijman S., Van Den Berg R., Ramkisoensing A., Boon M. R., Kuipers E. N., Loef M., Zonneveld T. C. M., Lucassen E. A., Sips H. C. M., Chatzispyrou I. A., Houtkooper R. H., Meijer J. H., Coomans C. P., Biermasz N. R., Rensen P. C. N. (2015). Prolonged
Daily Light Exposure Increases Body Fat Mass through Attenuation of
Brown Adipose Tissue Activity. Proc. Natl. Acad.
Sci. U.S.A..

[47] Tavolaro F. M., Thomson L. M., Ross A. W., Morgan P. J., Helfer G. (2015). Photoperiodic
Effects on Seasonal Physiology, Reproductive Status and Hypothalamic
Gene Expression in Young Male F344 Rats. J.
Neuroendocrinol..

[48] Shoemaker M. B., Heideman P. D. (2002). Reduced Body Mass, Food Intake, and Testis Size in
Response to Shortphotoperiod in Adult F344 Rats. BMC Physiol..

[49] Iglesias-Carres L., Mas-Capdevila A., Bravo F. I., Arola L., Muguerza B., Arola-Arnal A. (2019). Exposure of
Fischer 344 Rats to Distinct Photoperiods
Influences the Bioavailability of Red Grape Polyphenols. J. Photochem. Photobiol. B.

[50] Mariné-Casadó R., Domenech-Coca C., del Bas J. M., Bladé C., Arola L., Caimari A. (2018). Intake of an Obesogenic Cafeteria
Diet Affects Body Weight, Feeding Behavior, and Glucose and Lipid
Metabolism in a Photoperiod-Dependent Manner in F344 Rats. Front. Physiol..

