# Supplementary material for: Circannual Rhythms Affect the Bioavailability of Phenolic Compounds from Grape Seed Proanthocyanidins Extract Differently in Healthy and Obese Fischer 344 Rats
Source: J Agric Food Chem. 2025 Sep 26;73(40):25329–39. doi: 10.1021/acs.jafc.5c03952 (PMC12512188; doi:10.1021/acs.jafc.5c03952)
Supplement: Supplementary file 1 [file jf5c03952_si_001.pdf]

Supporting Information

Circannual rhythms affect the bioavailability of phenolic compounds from grape seed proanthocyanidins extract differently in healthy and obese Fischer 344 rats

Iván Escobar-Martínez, Verónica Arreaza-Gil[$], Anna Arola-Arnal, Begoña Muguerza, Miquel Mulero, Manuel Suárez* and Cristina Torres-Fuentes.

[1]*Universitat Rovira i Virgili, Departament de Bioquímica i Biotecnologia, Nutrigenomics Research Group, 43007 Tarragona, Spain*

[2]*Nutrigenomics Research Group, Institut d'Investigació Sanitària Pere Virgili (IISPV). C/ Marcel·lí Domingo 1, 43007 Tarragona, Spain*

[3]*Center of Environmental Food and Toxicological Technology (TecnATox). C/ Marcel·lí Domingo 1, 43007 Tarragona, Spain*

[$]*Current affiliation: Disease Biomarkers and Molecular Mechanisms Group (DIBIOMEC), Institut d'Investigació Sanitària Pere Virgili (IISPV), C/Dr. Mallafré Guash, 4, 43005 Tarragona, Spain*

*Corresponding author: manuel.suarez@urv.cat

Table S1: Optimized MRM transitions for detection of phenolic metabolites in rat serum.

Compound	MW	RT (min)	MS/MS quantification		MS/MS confirmation	
			MRM_1	CE (V)	MRM_2	CE(V)
(+)-Catechin	290.27	4.8	289.1 > 203	12	289.1 > 245	10
Procyanidin dimer B2	578.52	4.9	577.5 > 407	24	577.5 > 425	16
3,4,5-Trihydroxybenzoic acid	170.12	2.7	169 > 125	12	169 > 79	28
4-Hydroxy-3-methoxybenzoic acid	168.15	3.8	167 > 123	12	167 >108	20
3-(4'-hydroxyphenyl) propanoic acid	182.17	5.6	181 > 163	10	181 > 134	20
3'-hydroxyphenylacetic acid	152.15	2.5	151 > 107	12	151 > 93.1	20
Hippuric acid	179.17	4.2	178 > 134	8	178 > 132	16
3',4'-Dihydroxycinnamic acid	180.16	4.2	179 > 135	16	179 > 107	24
4'-Hydroxy-3'-methoxycinnamic acid	194.18	6.5	193 > 193	0	193 > 175	10
Benzoic acid	122.12	6.3	121 > 77	8	121 > 59	4
3-Hydroxybenzoic acid	138.12	4.6	137 > 65	36	137 > 93	20

All compounds were analyzed in negative ion mode. Fragmentor used in all compounds was 380 V. *Abbreviations*: MW, Molecular weight; RT, retention time; MRM, Multiple Reaction Monitoring; CE, collision energy; V, Volts.

Table S2: Method validation parameters (calibration, LOD, LOQ).

Compound	Calibration curve	R^2	Linearity (ppb)	LOD (ppb)	LOQ (ppb)
(+)-Catechin	y = 41.98x	0.991	23-5750	1.812	6.039
Procyanidin dimer B2	y = 32.21x	0.997	15-3750	0.445	1.482
3,4,5-Trihydroxybenzoic acid	y = 331.87x	0.998	28-3500	1.316	4.386
4-Hydroxy-3-methoxybenzoic acid	y = 16.01x	0.997	22-2750	24.665	82.217
3-(4'-hydroxyphenyl) propanoic acid	y = 19.03x	0.996	20-2500	2.132	7.106
3'-hydroxyphenylacetic acid	y = 3.01x	0.975	25-3125	21.33	71.101
Hippuric acid	y = 163.28x	0.998	27-3375	2.635	8.782
3',4'-Dihydroxycinnamic acid	y = 7.39x	0.995	20-2500	33.574	111.912
4'-Hydroxy-3'-methoxycinnamic acid	y = 148.6x	0.997	22-2750	1.037	3.457
Benzoic acid	y = 53.60x	0.986	22-2750	1.703	5.678
3-Hydroxybenzoic acid	y = 253.87x	0.998	40-5000	12.275	40.917

Abbreviations: RT, retention time; R^2, determination coefficient; LOD, limit of detection; LOQ, limit of quantification.

Table S3: Phenolic metabolite concentrations in vehicle-treated rats.

**VH** Compound	Standard Diet (ST)			Cafeteria Diet (CAF)		
	L6	L12	L18	L6	L12	L18
**Σ Flavan-3-ols**	**0.01 ± 0.001**	**n.d.**	**0.007 ± 0.004**	**n.d.**	**n.d.**	**n.d.**
(+)-Catechin	0.01 ± 0.001	n.q.	0.007 ± 0.004	n.q.	n.q.	n.d.
(–)-Epicatechin	n.d.	n.d.	n.d.	n.q.	n.q.	n.q.
Procyanidin dimer B1	n.d.	n.d.	n.d.	n.d.	n.d.	n.d.
Procyanidin dimer B2	n.d.	n.d.	n.d.	n.d.	n.d.	n.d.
3,4,5-Trihydroxybenzoic acid	n.d.	n.d.	n.d.	n.d.	n.d.	n.d.
4-Hydroxy-3-methoxybenzoic acid	n.d.	n.d.	n.d.	n.d.	n.d.	n.d.
**Σ Phase-II flavan-3-ols**	**1.658 ± 0.708**	**1.981 ± 0.73**	**3.244 ± 1.687**	**0.2 ± 0.131**	**0.18 ± 0.071**	**0.528 ± 0.22**
(+)-Catechin gluc [b]	0.419 ± 0.301	0.538 ± 0.405	0.923 ± 0.533	n.d.	n.d.	n.q.
(–)-Epicatechin gluc [c]	n.d.	n.q.	n.q.	n.d.	n.d.	n.d.
Methyl-catechin gluc [b]	0.294 ± 0.169	0.185 ± 0.074	0.882 ± 0.854	0.014 ± 0.018	0.035 ± 0.044	0.097 ± 0.06
Methyl-epicatechin gluc [c]	0.941 ± 0.38	1.25 ± 0.444	1.433 ± 0.556	0.184 ± 0.117	0.143 ± 0.055	0.428 ± 0.211
Catechin sulphate [b]	n.d.	n.d.	n.d.	n.d.	n.d.	n.d.
Epicatechin sulphate [c]	n.d.	n.d.	n.d.	n.d.	n.d.	n.d.
3-*O*-methylgallic acid	0.004 ± 0.003	0.007 ± 0.004	0.005 ± 0.002	0.002 ± 0.001	0.002 ± 0.003	0.002 ± 0.001
3-*O*-Methyl epicatechin [c]	n.d.	n.d.	n.d.	n.d.	n.d.	n.d.
4-*O*-Methyl epicatechin [c]	n.d.	n.d.	n.d.	n.d.	n.d.	n.d.
Methyl-cate/epi sulphate [b,c]	n.d.	n.d.	n.d.	n.d.	n.d.	n.d.
**Σ Microbial metabolism**	**128.837 ± 11.918**	**120.207 ± 34.718**	**130.577 ± 20.502**	**62.794 ± 12.04**	**72.762 ± 9.865**	**64.811 ± 10.923**
Phenylacetic acid	11.525 ± 4.409	12.363 ± 3.272	9.815 ± 5.103	4.432 ± 1.631	4.789 ± 1.542	3.145 ± 1.437
3-(4'-hydroxyphenyl) propanoic acid	0.093 ± 0.041	0.184 ± 0.087	0.305 ± 0.238	0.244 ± 0.243	0.536 ± 0.392	0.113 ± 0.174
3',4'-dihydroxyphenylacetic acid	n.d.	n.d.	n.d.	n.d.	n.d.	n.d.
3'-hydroxyphenylacetic acid	2.155 ± 0.683	2.375 ± 0.461	2.004 ± 0.862	0.877 ± 0.397	0.811 ± 0.153	0.946 ± 0.465
4'-hydroxyphenylacetic acid	n.d.	n.d.	n.d.	n.d.	n.d.	n.d.
4'-Hydroxy-3'-methoxyphenylacetic acid	47.077 ± 9.904	45.502 ± 3.807	47.552 ± 9.1	44.138 ± 7.192	55.968 ± 8.546	46.411 ± 6.753

Hippuric acid	33.913 ± 4.955	29.408 ± 16.281	34.055 ± 14.896	6.59 ± 3.497	5.035 ± 2.408	6.648 ± 2.262
3',4'-Dihydroxycinnamic acid	27.244 ± 4.918	23.324 ± 13.072	28.443 ± 12.353	5.02 ± 2.594	3.961 ± 1.815	6.237 ± 3.379
4'-Hydroxy-3'-methoxycinnamic acid	0.031 ± 0.011	0.038 ± 0.013	0.042 ± 0.012	0.016 ± 0.011	0.012 ± 0.003	0.014 ± 0.007
Benzoic acid	1.433 ± 0.134	1.428 ± 0.399	1.407 ± 0.192	1.476 ± 0.714	1.65 ± 0.461	1.299 ± 0.4
3-Hydroxybenzoic acid	n.q.	n.q.	n.q.	n.q.	n.q.	n.q.
Phenylpropionic Acid	5.367 ± 1.015	5.584 ± 2.796	6.955 ± 3.293	n.d.	n.d.	n.d.

Abbreviations: L6, short photoperiod (6 h light / 18 h dark); L12, standard photoperiod (12 h light / 12 h dark); L18, long photoperiod (18 h light / 6 h dark); not detected (n.d.); not quantified (n.q.); glucuronide (gluc). [a)] Quantified using the calibration curve of procyanidin dimer B2; [b)] Quantified using the calibration curve of catechin; [c)] Quantified using the calibration curve of epicatechin; [d)] Quantified using the calibration curve of 3,4,5-trihydroxybenzoic acid; [e)] Quantified using the calibration curve of 3'-hydroxyphenylacetic acid; [f)] Quantified using the calibration curve of 4-Hydroxy-3-methoxybenzoic acid; [g)] Quantified using the calibration curve of 3-(4'-hydroxyphenyl) propanoic acid. Results are expressed as μM ± SD (n=8). The significance level was $p < 0.05$.

Table S4: Phenolic metabolite concentrations in GSPE-treated rats .

**GSPE** Compound	Standard Diet (ST)			Cafeteria Diet (CAF)		
	L6	L12	L18	L6	L12	L18
**Σ Flavan-3-ols**	**0.04 ± 0.031**	**0.04 ± 0.022**	**0.046 ± 0.027**	**0.044 ± 0.044**	**0.018 ± 0.007**	**0.01 ± 0.007**
(+)-Catechin	0.01 ± 0.004	0.019 ± 0.004	0.013 ± 0.006	0.019 ± 0.018	0.01 ± 0.006	0.01 ± 0.007
(−)-Epicatechin	0.03 ± 0.03	0.021 ± 0.02	0.033 ± 0.028	0.025 ± 0.028	0.008 ± 0.007	n.q.
Procyanidin dimer B1	n.d.	n.d.	n.d.	n.d.	n.d.	n.d.
Procyanidin dimer B2	n.d.	n.d.	n.d.	n.d.	n.d.	n.d.
3,4,5-Trihydroxybenzoic acid	n.d.	n.d.	n.d.	n.d.	n.d.	n.d.
4-Hydroxy-3-methoxybenzoic acid	n.d.	n.d.	n.d.	n.d.	n.d.	n.d.
**Σ Phase-II flavan-3-ols**	**34.914 ± 17.062**	**37.985 ± 11.42**	**32.588 ± 13.696**	**14.93 ± 9.1**	**5.317 ± 1.991**	**4.632 ± 1.969**
(+)-Catechin gluc [b]	6.276 ± 4.446	6.455 ± 4.014	7.415 ± 4.275	2.633 ± 2.613	n.d.	n.d.
(−)-Epicatechin gluc [c]	9.577 ± 8.81	6.294 ± 4.213	7.17 ± 4.162	5.054 ± 5.233	n.d.	n.d.
Methyl-catechin gluc [b]	1.238 ± 0.792	2.017 ± 0.862	1.903 ± 1.072	0.384 ± 0.148	0.277 ± 0.196	0.209 ± 0.116
Methyl-epicatechin gluc [c]	16.915 ± 5.819	22.229 ± 6.327	15.315 ± 7.222	6.505 ± 1.71	4.79 ± 1.696	4.232 ± 1.839
Catechin sulphate [b]	n.d.	n.d.	n.d.	n.d.	n.d.	n.d.
Epicatechin sulphate [c]	n.d.	n.d.	n.d.	n.d.	n.d.	n.d.
3-*O*-methylgallic acid	0.039 ± 0.019	0.065 ± 0.029	0.054 ± 0.036	0.039 ± 0.023	0.036 ± 0.026	0.019 ± 0.007
3-*O*-Methyl epicatechin [c]	n.d.	n.d.	n.d.	n.d.	n.d.	n.d.
4-*O*-Methyl epicatechin [c]	n.d.	n.d.	n.d.	n.d.	n.d.	n.d.
Methyl-cate/epi sulphate [b,c]	0.869 ± 0.499	0.925 ± 0.081	0.731 ± 0.453	0.314 ± 0.16	0.214 ± 0.098	0.173 ± 0.104
**Σ Microbial metabolism**	**106.633 ± 22.81**	**133.209 ± 15.559**	**116.833 ± 52.279**	**73.689 ± 21.437**	**78.536 ± 16.349**	**62.372 ± 17.852**
Phenylacetic acid	8.965 ± 2.066	11.294 ± 1.263	6.349 ± 1.534	3.866 ± 0.73	5.056 ± 1.263	3.199 ± 1.657
3-(4'-hydroxyphenyl) propanoic acid	0.116 ± 0.11	0.137 ± 0.131	0.223 ± 0.103	0.365 ± 0.429	0.138 ± 0.141	0.2 ± 0.172
3',4'-dihydroxyphenylacetic acid	n.d.	n.d.	n.d.	n.d.	n.d.	n.d.
3'-hydroxyphenylacetic acid	1.748 ± 0.395	2.185 ± 0.478	1.699 ± 0.537	0.752 ± 0.189	1.285 ± 0.432	0.844 ± 0.122
4'-hydroxyphenylacetic acid	n.d.	n.d.	n.d.	n.d.	n.d.	n.d.
4'-Hydroxy-3'-methoxyphenylacetic acid	42.011 ± 6.847	52.123 ± 8.388	43.688 ± 8.481	61.282 ± 19.256	56.215 ± 9.192	43.122 ± 9.255
Hippuric acid	26.886 ± 11.156	32.665 ± 5.288	30.841 ± 22.21	3.525 ± 1.755	8.07 ± 7.242	7.783 ± 5.372

3',4'-Dihydroxycinnamic acid	21.369 ± 8.74	26.585 ± 3.791	26.264 ± 18.902	2.675 ± 1.398	6.273 ± 5.697	5.991 ± 4.19
4'-Hydroxy-3'-methoxycinnamic acid	0.018 ± 0.006	0.038 ± 0.021	0.037 ± 0.019	0.008 ± 0.005	0.009 ± 0.006	0.058 ± 0.059
Benzoic acid	1.261 ± 0.269	1.495 ± 0.335	1.218 ± 0.321	1.216 ± 0.139	1.49 ± 0.412	1.176 ± 0.244
3-Hydroxybenzoic acid	n.q.	n.q.	n.q.	n.q.	n.q.	n.q.
Phenylpropionic Acid	4.259 ± 2.035	6.686 ± 1.498	6.514 ± 5.062	n.d.	n.d.	n.d.

Abbreviations: L6, short photoperiod (6 h light / 18 h dark); L12, standard photoperiod (12 h light / 12 h dark); L18, long photoperiod (18 h light / 6 h dark); not detected (n.d.); not quantified (n.q.); glucuronide (gluc). [a)] Quantified using the calibration curve of procyanidin dimer B2; [b)] Quantified using the calibration curve of catechin; [c)] Quantified using the calibration curve of epicatechin; [d)] Quantified using the calibration curve of 3,4,5-trihydroxybenzoic acid; [e)] Quantified using the calibration curve of 3'-hydroxyphenylacetic acid; [f)] Quantified using the calibration curve of 4-Hydroxy-3-methoxybenzoic acid; [g)] Quantified using the calibration curve of 3-(4'-hydroxyphenyl) propanoic acid. Results are expressed as μM ± SD (n=8). The significance level was $p < 0.05$.

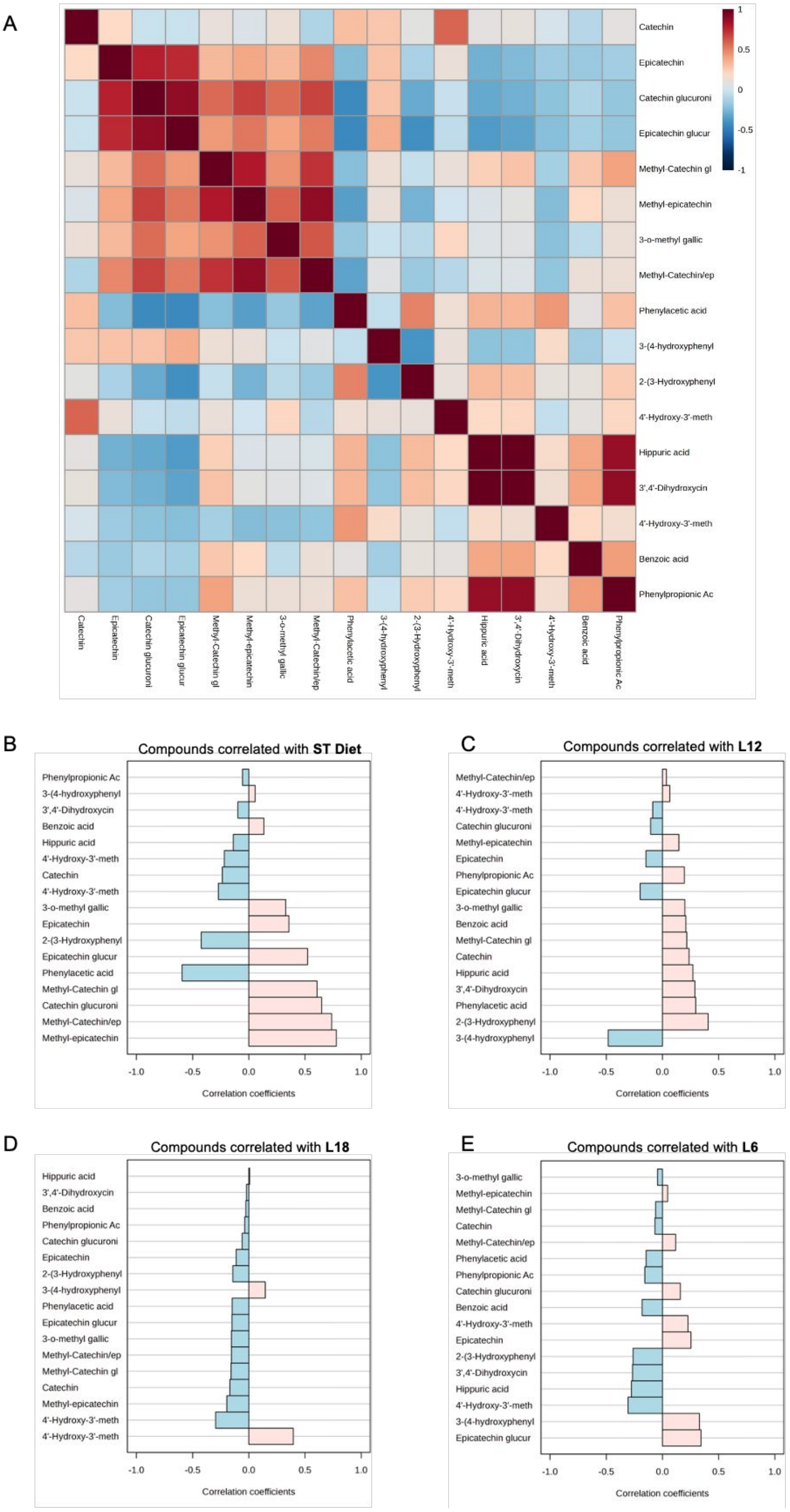
A
Catechin
Epicatechin
Catechin glucuroni
Epicatechin glucur
Methyl-Catechin gl
Methyl-epicatechin
3-o-methyl gallic
Methyl-Catechin/ep
Phenylacetic acid
3-(4-hydroxyphenyl
2-(3-Hydroxyphenyl
4'-Hydroxy-3'-meth
Hippuric acid
3',4'-Dihydroxycin
4'-Hydroxy-3'-meth
Benzoic acid
Phenylpropionic Ac
1
0.5
0
-0.5
-1
B
Compounds correlated with **ST Diet**
Phenylpropionic Ac
3-(4-hydroxyphenyl
3',4'-Dihydroxycin
Benzoic acid
Hippuric acid
4'-Hydroxy-3'-meth
Catechin
4'-Hydroxy-3'-meth
3-o-methyl gallic
Epicatechin
2-(3-Hydroxyphenyl
Epicatechin glucur
Phenylacetic acid
Methyl-Catechin gl
Catechin glucuroni
Methyl-Catechin/ep
Methyl-epicatechin
-1.0
-0.5
0.0
0.5
1.0
Correlation coefficients
C
Compounds correlated with **L12**
Methyl-Catechin/ep
4'-Hydroxy-3'-meth
4'-Hydroxy-3'-meth
Catechin glucuroni
Methyl-epicatechin
Epicatechin
Phenylpropionic Ac
Epicatechin glucur
3-o-methyl gallic
Benzoic acid
Methyl-Catechin gl
Catechin
Hippuric acid
3',4'-Dihydroxycin
Phenylacetic acid
2-(3-Hydroxyphenyl
3-(4-hydroxyphenyl
-1.0
-0.5
0.0
0.5
1.0
Correlation coefficients
D
Compounds correlated with **L18**
Hippuric acid
3',4'-Dihydroxycin
Benzoic acid
Phenylpropionic Ac
Catechin glucuroni
Epicatechin
2-(3-Hydroxyphenyl
3-(4-hydroxyphenyl
Phenylacetic acid
Epicatechin glucur
3-o-methyl gallic
Methyl-Catechin/ep
Methyl-Catechin gl
Catechin
Methyl-epicatechin
4'-Hydroxy-3'-meth
4'-Hydroxy-3'-meth
-1.0
-0.5
0.0
0.5
1.0
Correlation coefficients
E
Compounds correlated with **L6**
3-o-methyl gallic
Methyl-epicatechin
Methyl-Catechin gl
Catechin
Methyl-Catechin/ep
Phenylacetic acid
Phenylpropionic Ac
Catechin glucuroni
Benzoic acid
4'-Hydroxy-3'-meth
Epicatechin
2-(3-Hydroxyphenyl
3',4'-Dihydroxycin
Hippuric acid
4'-Hydroxy-3'-meth
3-(4-hydroxyphenyl
Epicatechin glucur
-1.0
-0.5
0.0
0.5
1.0
Correlation coefficients

Figure S1. Correlation analysis between metabolite profiles, diet, and photoperiod. (**A**) A correlation heatmap displays Pearson r coefficients representing associations between various metabolites, with red indicating positive correlations and blue indicating negative ones. (**B, C, D, E**) Correlation analyses, conducted using Pattern Hunter (Pearson r), between diet groups and photoperiod conditions, are visualized in these panels. In each panel, the color scheme remains consistent, with red indicating positive correlations and blue representing negative associations. Note: The abbreviation "4′-hydroxy-3′-meth" corresponds to 4′-hydroxy-3′-methoxyphenylacetic acid (first) and 4′-hydroxy-3′-methoxycinnamic acid (second), following the same order as in the main tables.
